# Initial Feasibility and Criterion Validation of an Electronic, Self-Administered, Geriatric Assessment to Facilitate Fitness-To-Drive Evaluations in Older Adults

**DOI:** 10.20900/agmr20260011

**Published:** 2026-05-18

**Authors:** Luke D. Braun, William P. Gahan, Johnathon Ehsani, Michelle Duren, Sara Seifert, Louis Barrett, Kevin Kramer, Jeffrey N. Keller

**Affiliations:** 1 Pennington Biomedical Research Center, 6400 Perkins Road, Baton Rouge, LA 70808, USA; 2 Johns Hopkins Bloomberg School of Public Health, Baltimore, MD 21205, USA; 3 Minnesota HealthSolutions Corporation, Minneapolis, MN 55414, USA

**Keywords:** driving safety, fitness-to-drive, geriatric assessment, older adults, telehealth, electronic health assessment

## Abstract

**Background:**

Geriatric assessments are considered the gold standard for the medical evaluation and management of older adult patients’ even though the time required for administration, and additional logistical challenges, has impeded widespread adoption in clinical care.

**Objective:**

The current study examines the initial feasibility and criterion validity of a brief, electronic, self-guided (BES) geriatric assessment designed to capture multiple medical and non-medical aspects of a comprehensive geriatric assessment including informing medical fitness-to-drive determinations.

**Methods:**

Data was obtained from 49 older adult drivers (34 female, 15 male) aged 50–85. Feasibility was evaluated by determining the ability of older adult drivers to complete the BES geriatric assessment remotely without assistance. Criterion validity was assessed by comparing self-reported responses on the BES geriatric assessment to their responses during a subsequent clinician-led consultation with a geriatrician. Validity was operationalized as concordance (proportion of items requiring no correction) and comprehensiveness (frequency and nature of supplementary details elicited during the clinical interview).

**Results:**

Our results support the BES geriatric assessment being a feasible and valid mechanism for the collection of medical and non-medical data related to a comprehensive geriatric assessment used as part of a fitness-to-drive evaluation. All 49 participants successfully completed the BES geriatric assessment without assistance, supporting feasibility of self-administration in this population. Self-reported digital responses demonstrated concordance with clinician-verified data (accuracy: 100%, 95% CI: 92.7%–100%). The geriatrician-led consultation elicited 41 supplementary items across 26 participants (53.1%) that provided additional contextual detail to reported events. All contextual clarifications were consistent with open-ended clinical interviewing, with no contradicted self-reported data reported.

**Conclusions:**

The BES geriatric assessment represents a feasible and accurate approach for collecting comprehensive geriatric assessment level data in older adult drivers. This approach has the potential to increase patient access to comprehensive geriatric assessments and streamline medical fitness-to-drive determinations.

## INTRODUCTION

Geriatric assessments are considered the gold standard for the medical evaluation and management of evolving health conditions observed in older adult patients [1,2] due to the multidisciplinary and comprehensive approach used to capture vital medical, cognitive, social, functional, and non-medical patient data. This approach is particularly important in the context of older adults who have two or more chronic medical conditions that are actively evolving and impacting activities of daily living (ADLs) and/or instrumental ADLs (iADLs) [1,3]. Understanding changes in the ability of the patient to support their ADLs (bathing, grooming, etc.) and iADLs (maintaining home, transportation, etc.) are central to making determinations on the independence and safety of the patient. Studies have demonstrated that use of a geriatric assessment as part of patient care improves health outcomes, increases patient quality-of-life (QoL), and improves care coordination [4-7]. Despite the numerous documented benefits, geriatric assessments are frequently not provided to patients because of the real/perceived demands of being burdensome, time consuming, and challenging to coordinate in the clinical workflow [8]. Developing clinical approaches that lower the clinical burden for geriatric assessment administration is an important and active area of clinical research [9].

Specialized geriatric assessments have been developed for patients with specific medical conditions [1,2]. These efforts facilitate the ability of health providers to create a care plan that accounts for the mitigation of adverse events associated with evolving health conditions, and potential adverse treatment effects, while also ensuring patient goals and priorities [1,2].

Driving is routinely reported as being one of the most important activities for older adults to maintain their independence and QoL [10]. Medical decision-making related to potential restrictions, suspensions, or termination of driving privileges in older adult patients is routinely cited as one of the most challenging aspects of healthcare delivery [11-14]. Healthcare providers frequently cite having a lack of guidance and insufficient data when making fitness-to-drive decisions in their older adult patients [13,14]. Multiple states have implemented reporting rules for healthcare providers caring for patients that may be unsafe to drive [15], with the presence of a medical condition frequently insufficient to use as the sole proxy for fitness-to-drive determinations [16]. Lastly, cessation of driving is known to increase all-cause morbidity and decrease the QoL for older adult patients [17]. Together, these data identify the potential benefit for incorporating geriatric assessments as part of medical fitness-to-drive evaluation process in older adults to help prevent the implementation of inappropriate driving restrictions.

Several studies have identified utility in using abbreviated, electronic, and/or self-guided geriatric assessments as a mechanism to expand their implementation in diverse care settings [8,18]. The current study extends the impact of these previous clinical research efforts by evaluating the feasibility of using a brief, electronic, and self-guided (BES)-geriatric assessment to collect medical and non-medical data, including data to inform medical fitness-to-drive determinations. Tools like the BES-geriatric assessment could therefore increase the ability of older adults and their stakeholders (families/physicians) to have discussions on continuing or discontinuing/restricting driving that are informed based on patient data that otherwise is not able to be collected currently, and subsequently aid physicians in making more informed decisions related to driving safety in their older adult patients. It is important to note that the BES geriatric assessment, like all other brief electronic geriatric assessments, does not currently include functional assessments and is intended to facilitate (and not replace) in-clinic functional evaluations. The BES geriatric assessment and geriatrician-led consultations outlined herein were conducted as part of a clinical research study investigating the impact of combining smartphone-based assessments (BES-geriatric assessment) and real-world driving data (smartphone telematics) to support safer driving in 49 older adult drivers. Initial feasibility was evaluated by determining the ability of the 49 older adult drivers to complete the assessment in an independent and self-guided manner and criterion validity quantified by the concordance and comprehensiveness of responses on the BES-geriatric assessment to the responses obtained in subsequent physician-led consultations.

## MATERIALS AND METHODS

### Study Participants

The data outlined herein were obtained as part of a National Institutes of Health funded study examining the feasibility of using an App-based approach with older adult drivers to collect driver health information, real-world driving data (telematics), and provide customized driving feedback to reduce risky driving behaviors. A total of 51 older adult drivers aged 50–85 was enrolled in the main study between 2025 February 17 and 2025 March 19. Participants were compensated $50 for completion of the 12-week main study. A total of 49 participants enrolled in the geriatrician consultation effort described herein. Some participants also participated in coaching sessions (42 participants) and exit surveys (44 participants) which are both part of a separate manuscript submission reporting on the functionality of the driving app. All participants provided written informed consent prior to the initiation of any study procedures. All study procedures were approved by, and conducted in accordance with, the Johns Hopkins University Institutional Review Board (Study # 28992/MOD5807).

All participants were recruited from an ongoing longitudinal study of brain aging [19,20]. Participants who authorized being contacted about future studies were sent a general interest email that contained brief information about the study and eligibility criteria (age, smartphone access, minimal driving requirements). Participants with a diagnosis of any form of dementia, or performance on cognitive tests during the longitudinal study consistent with the presence of dementia, were excluded from participation in the current study. Interested participants received an introductory email and electronic consent. Participants received an email allowing them to access the BES geriatric assessment.

### Brief, Electronic, Self-Guided Geriatric Assessment

The BES geriatric assessment was created to provide a customizable, rapid, and low-burden mechanism for the capture of multiple medical and non-medical aspects of a comprehensive geriatric assessment in order to inform medical fitness-to-drive efforts. The BES geriatric assessment collects data on driver demographics, health history, medication profile, current living situation, access to transportation, driving history, perceptions on driving, and current driving self-restrictions (where applicable). The specific components for each of these categories is briefly outlined below. Within 2 weeks of completing the BES geriatric assessment participants underwent a physician-led consultation (virtual consultation) with a geriatrician (MD). The same geriatrician completed all consultations with all 49 study participants. The participants were scheduled for the geriatrician consultation via calls/emails with study staff following completion of the BES geriatric assessment. Each component of the BES geriatric assessment was re-examined during the geriatrician-led consultation to allow for comparison of responses during physician-led versus self-guided assessment.

Demographics: age, gender (male, female, other), race (American Indian/Alaskan native, Asian, Native Hawaiian, black/African American, white, more than one race, other), ethnicity (Hispanic or Latino, non-Hispanic/Latino, other, prefer not to say), marital status, education, zip code.

Living situation and access to transportation: live alone (yes, no), home environment (urban, rural, other), access to a reliable vehicle (if yes, the number of vehicles accessible), access to other drivers in the home (if yes then list number), access to alternative modes of transport (public transportation, volunteer ride share, paid rideshare, family/friends, other).

Driving history: recent moving violations (last 5 years), recent accidents (last 5 years), driving under influence or driving while impaired (yes, no), description of roads driven (interstate, freeway, highway, scenic highway, neighborhood, other), knowledge of driving rules in state (excellent, very good, good, fair, poor), easily distracted (yes, no), know if vehicle has advanced driver assist system (If yes do you use it, if yes do you feel it makes you a safer driver).

Perceptions of driving abilities (for each of following choices the options are excellent, very good, good, fair, poor relative to others your age): perceived driving abilities, ability to turn head while driving, mobility, reflexes related to driving, vision, hearing, ability to concentrate.

Perceived importance of driving (for each of the following choices are very important, somewhat important, not very important, not important): independence, QoL, overall health.

Driving self-restrictions: self-restrictions on the amount of driving in last 2 years (yes, no), self-restrictions on driving in specific scenarios (if yes has restriction made life more challenging), type of self-restriction (rush hour, highway/interstate, inclement weather, nighttime, construction, parking lots, new routes, congested areas), cell phone use (if yes there is free text box to describe what cell phone is used for).

Health related to driving: overall health relative to drivers your age (excellent, very good, good, fair, poor), hospitalized in the last 2 years (if yes how long ago), surgery with sedation/anesthesia in last 2 years (if yes how long ago), fallen in the last 2 years (if yes how many times), use of any walking aids on a regular basis (yes, no), frequent urinary urgency (yes, no), frequent daytime drowsiness (if yes rate drowsiness as not a concern, somewhat of a concern, a concern, significant, very significant), use of dialysis (yes, no), use prescription glasses (yes, no), diagnosed with any of following vision-related conditions (cataracts, diabetic retinopathy, farsightedness, glaucoma, macular degeneration, nearsightedness, other), had any of the following (cataract surgery, eye injections, glaucoma surgery, Lasik surgery, prescription glasses, strabismus surgery), arthritis (if yes which areas are affected: ankles, back, feet, hands, knees, neck, wrist).

Which of the following medical conditions have you been diagnosed with (if yes describe): anxiety disorder, arthritis, attention deficit hyperactivity disorder, autism spectrum disorder, cancer, cognitive impairment, congestive heart failure, dementia, depression, diabetes, epilepsy, glaucoma, high blood pressure, high cholesterol, hearing loss, heart disease, kidney/renal disease, long COVID, macular degeneration, mild cognitive impairment, neuropathy, Parkinson’s disease, peripheral vascular issue, respiratory issue, retinopathy, shortness of breath, sleep apnea, sleep disorder other than sleep apnea, psychiatric condition, stroke, traumatic brain injury, other.

Have you spoken with your physician about your driving (if yes rate as helpful or not helpful).

Medications: do you take 5 or more prescribed medications on a daily basis (yes, no), how many prescribed medications do you take on a daily basis, do you take any of the following medications (allergy medications, anticonvulsants, anti-anxiety medications, antidepressants, barbituates, benzodiazapines, diabetes medications, eye medications, muscle relaxers, NSAIDs, over active bladder medications, pain medications, Parkinsons medications, sleep medications), which over-the-counter medications do you take (free text box).

### Statistical Analysis

Feasibility was evaluated as the proportion of participants who successfully completed the BES geriatric assessment without technical- or content-related assistance. Criterion validity was assessed through two complementary metrics, concordance and comprehensiveness. Concordance was defined as the proportion of self-reported items requiring no correction during subsequent physician-led consultation and comprehensiveness defined as the frequency and nature of supplementary details obtained during the physician-led consultation. Both feasibility and validity outcomes were calculated with 95% confidence intervals using the Wilson score method, which provides accurate coverage for extreme variance (0%–100%) in small-to-moderate sample sizes [21]. The descriptive statistics for comprehensiveness measures included total number of elaborations identified.

## RESULTS

In addition to reporting on the initial feasibility and validity of the BES geriatric assessment, the data from the current study is intended to demonstrate the breadth and actionable nature of the data collected as part of a BES geriatric assessment.

### Participant Demographics

The 49 participants in the current study sample were predominantly female (34, 69.4%), white (46, 94%), and non-Hispanic (48, 98%). The mean and standard deviation of participants was 68 (±8.2 years). A total of 38 (78%) of the participants were married, 4 were divorced (8.1%), 3 were widowed (6.1%), and 4 were single (8.1%).

### Feasibility and Criterion Validity

Feasibility was evaluated by assessing the ability of the participants to complete the BES geriatric assessment without technical or content-related assistance. In the current study we observed 100% feasibility with all participants completing the BES geriatric assessment in a self-guided and remote manner without any assistance from the study staff. Criterion validity was evaluated using concordance (self-reported responses needing no correction) and comprehensiveness (frequency and nature of new supplementary information obtained during physician-led consultation). The self-reported digital responses demonstrated strong concordance with clinician-verified data (accuracy: 100%, 95% CI: 92.7%–100%). The geriatrician-led review elicited 41 supplementary items across 26 participants (53.1% of participants) that provided additional contextual detail to self-reported events. The contextual clarifications obtained were all consistent with open-ended clinical interviewing, with no contradicted self-reported data reported.

The new data captured as part of the geriatrician-led consultation obtained new details and clarifications in the areas of accidents, moving violations, surgeries, treatments, medications, treatment modifications, and/or falls reported on the BES geriatric assessment. For example, drivers highlighted for some of the accidents whether they were at fault, in the context of DUIs the drivers indicated how many decades previously the violation occurred, and with falls the reasons for the fall was identified. Similarly, for obstructive sleep apnea (OSA) some drivers provided clarification they do utilize continuous positive airway pressure (CPAP) therapy, while others clarified that the OSA no longer required treatment because of significant amounts of weight loss. Together, these data identified the need to add free text boxes and/or incorporating an additional branched logic question design within the BES geriatric assessment in order to more completely capture relevant patient data.

### Brief Summary of BES Geriatric Assessment Findings

Detailed data tables outlining specific findings with the BES geriatric assessment in the current study are provided as a supplementary data file (Tables S1-S5). Together these tables demonstrate the ability of the BES geriatric assessment to collect data vital to a geriatric assessment and fitness-to-drive determination. Data collected included relevant patient health history, medications, driving history, medical contributors to driving risk, perceptions of driving, and infrastructure to support alternative transportation options. A brief summary of BES geriatric assessment findings is provided below.

### Health Conditions Related to Driving

Most participants (38, 80%) reported their health as being very good to excellent, with nearly all participants (44, 90%) reported having at least 1 chronic medical condition known to adversely impact driving risk. The majority of participants (33, 67%) had 2 or more chronic medical conditions known to adversely impact driving and 11 (22.4%) reported having 4 or more chronic medical conditions. The most reported medical conditions included arthritis (16, 33%), depression (12, 24%), hearing loss (10, 20%), sleep apnea (9, 18%), cancer (9, 18%), chronic pain (3, 6%), glaucoma (3, 6%), general anxiety disorder (3, 6%), and heart disease (3, 6%). A total of 9 participants reported daytime drowsiness.

Most participants reported hearing (32, 65%), vision (26, 53%), and mobility (43, 88%) to be very good to excellent. Only 2 participants (4%) reported using walking aids. There were 9 participants (18%) who reported having a fall in the last 2 years, with 4 of these participants reporting more than 1 fall. A total of 8 participants (16%) reported being hospitalized in the last 2 years and 20 (41%) reported having surgeries in the last 2 years.

### Medications

Older adult drivers are particularly susceptible to have their driving negatively impacted when 5 or more prescribed medications are taken regularly (polypharmacy) [22,23]. The susceptibility to adverse impacts on driving and increased crash risk with polypharmacy in OA drivers is based in part on changes in metabolism, the presence of underlying medical issues, and increased susceptibility to potentially adverse drug-drug interactions [22,23]. A total of 15 participants (31%) reported taking 5 or more medications daily (polypharmacy). A total of 41 prescribed medications that are known to potentially negatively impact driving safety were reported being taken by the study participants. Included in these high-risk medications were allergy medications (19, 39%), pain medications (8, 16%), anti-depressants (8, 16%), diabetes medications (7, 14%), sleep aids (6, 12%), overactive bladder treatments (3, 6%) and muscle relaxers (2, 4%).

### Current Living Situation and Access to Transportation

The vast majority of participants lived with their spouse or life partner (40, 82%) and resided in an urban environment (39 participants, 80%). Most participants (34, 69%) reported having additional drivers in the home. All participants reported having access to reliable vehicle. Most of the participants drove a vehicle that was 3 years of age or older (28, 57%). A significant number of drivers reported their vehicle was equipped with an advanced driver assist systems (ADAS) in their vehicle (26, 53%) and each of these individuals reported that they believed use of ADAS made them a safer driver. The majority of the participants reported having access to alternative modes of transportation (31, 63%). The most reported alternative modes of transportation reported were family members (26, 67%), paid rideshare services (9, 18%), and public transportation (6, 12%).

### Daily Driving Patterns, Accidents, and Moving Violations

Nearly all drivers drove 5 or more days a week (42, 86%). Diversity was seen in the types of roads regularly driven with 34 participants reporting to regularly drive the interstate (69%), 35 regularly driving on the highway (71%), and 43 regularly driving in neighborhoods (88%). A total of 29 participants (59%) reported regularly driving on at least 3 different types of roads. A small number of participants (7, 14%) reported having a previous accident in the last 2 years. A similar number, but separate group of drivers (8, 16%) reported receiving moving violations in the last 2 years. Two participants (4%) reported having their license suspended previously and 2 participants (4%) reported being charged with driving under the influence (DUI) in the distant past.

### Perceptions of Driving

The majority of participants (44, 90%) rated their driving as being very good to excellent. A significant number of participants (21, 43%) reported using their phone while driving. The most common reasons provided for using the phone while driving were to conduct calls/text (18, 37%), to assist with navigation (9, 18%), and to listen to podcast/music (4, 8%). Most participants (41, 84%) reported that their ability move their head, neck, shoulders was very good to excellent. A similar number of participants (44, 90%) reported their knowledge of roadway rules to be very good to excellent. A smaller number of participants (39, 80%) reported their reflexes as being very good to excellent. Nearly all (47, 95%) reported that driving was very important or essential to maintaining their independence, QoL (45, 92%), and overall health (42, 86%).

### Driving Self-Restrictions and Discussions of Driving with a Physician

A total of 9 (18%) participants reported that they have intentionally restricted the amount they drive in the last 2 years. A large number of participants reported regularly restricting the places or conditions in which they drive (33, 67%) with the most common situations being inclement weather (22, 45%), avoiding congestion (20, 41%), not driving at night (22, 45%), avoiding rush hour traffic (21, 43%), and not driving at sites with ongoing construction (3, 6%). Only 2 participants (4%) reported having conversations with their physician about their driving with both participants stating that those conversations were helpful.

## DISCUSSION

The current study demonstrates the feasibility of using the BES geriatric assessment to capture a significant portion of the medical and non-medical data necessary for a comprehensive geriatric assessment and/or medical fitness-to-drive evaluation. Specifically, we demonstrate the utility of using this remote and self-guided approach in older adults to collect driver health history relevant to driving safety, medications, relevant driving history, current perceptions related to driving, access to alternative modes of transportation, and driving self-restrictions (if any). These findings raise the potential of incorporating a low-burden BES geriatric assessment as part of a multipronged effort designed to mitigate the current clinical burden of conducting comprehensive geriatric assessments and medical fitness-to-drive assessments. In this model, the findings of the BES geriatric assessment could be used to inform and focus subsequent in-clinic examinations and/or on-road evaluations by efficiently providing health professionals (physicians, occupational therapists) key medical and non-medical findings in advance of a clinic visit. Additionally, the use of the BES geriatric assessment would be expected to provide patients and their caregiver more flexibility as to when and where they provide health professionals the information they need, and thereby lower overall patient burden.

A unique feature of the BES geriatric assessment is that it not only collects information on driver health and driving, but it also captures impactful contextual information for each of these components. For example, while establishing the profile of chronic medical conditions (sleep apnea, chronic pain, arthritis, etc.) the BES geriatric assessment also collects data on the drivers’ perceptions of disease severity and their current perception of their physical abilities relevant to driving (thinking, memory, reflexes, range of motion, vision, hearing, etc.). Similarly, in addition to capturing data on driving habits and access to alternative modes of transportation the BES geriatric assessment also captures the perceived importance of driving to their independence, QoL, and overall health. The assessment also identifies self-restrictions in driving that are critical to accurately understanding evolutions in driving behavior. Lastly, the assessment captures important information on their perceived knowledge of traffic regulations and levels of distractibility.

Fitness-to-drive determinations primarily occur in reaction to newly emergent or rapidly worsening health conditions and/or recent compromises in driving safety. Only a small percentage of older adults in this study, and other studies [24,25], report having discussed driving with their physician. The paucity of proactive and early discussions between physicians and their patients about driving is the result of numerous factors including time constraints, physician discomfort, and a lack of data to inform decision-making [24,25]. The relatively low burden and dynamic nature of the BES geriatric assessment may be able to facilitate proactive physician-patient discussions on driving in the future by creating low-burden regular reports to facilitate and guide discussions between the physician and patient on this topic.

Access to behind-the-wheel evaluations is extremely limited nationwide and is not a viable option for a majority of older adult drivers. Behind-the-wheel testing is also frequently unable to capture early transitions from low-risk to high-risk driving, may not accurately capture day-to-day driving performance, and does not fully capture the range of environments a driver is exposed to on the road [26,27]. These realities have led to a call for using a tiered approach to inform medical fitness-to-drive determinations whereby surrogate measures are used to inform physicians about the driving risk/performance of their older adult patients and reserve behind-the-wheel testing for patients in the greatest need for such evaluations [24,28,29]. Utilization of the BES geriatric assessment, in combination with surrogate measures to behind-the-wheel evaluations, may aid in the creation of multiple validated and tiered options that allow health providers to accurately measure driving risk and changes in driving performance independent of behind-the-wheel testing. Similarly, while guidelines from the American Medical Association and the National Highway Traffic Safety Administration have been helpful in improving clinical care and roadway safety for older adult drivers, they have provided limited specificity to guide risk/benefit determinations. Tools like the BES geriatric assessment may play an important role in the future to aid health providers in more readily being able to parse evolving changes in driver health and driving behaviors into an actionable framework that provides firm stratifications of driver risk combined with clear care recommendations.

The current study identified several quick modifications that can be made to increase the impact of the BES geriatric assessment including the use of free text boxes and branched logic to capture clarifications and contextual information that is not currently being captured, need to create a media file summary for incorporation into electronic health records, as well as adding questions related to risk factors not examined in the current study (alcohol use, cannabis use, etc.). Lastly, based on the experiences of the current study we will be revising the BES to also include in the report relevant recommendations/guidelines for each observed health/driving risk.

The use of telematics has emerged as a leading-edge technology for the capture of real-world driving, quantifying risky driving behaviors (RDBs), and understanding overall driving risk [30]. The vast majority of telematics currently in use employs the GPS and related sensors in smartphones to measure a vast array of RDBs including speeding, hard braking, hard acceleration, and distracted driving [30]. Because the BES geriatric assessment can be administered via a smartphone App it is likely that it can be paired with a smartphone-based telematics platform to provide even more robust and impactful data to health professionals.

The potential for artificial intelligence (AI) agents to improve the stakeholder experience is multifaceted and ranges from having the AI agent being able to support robust interactions with stakeholders seeking additional information/clarification, to the development of predictive modeling for both driver health and roadway safety. In this model, a range of AI functionality could be incorporated to inform risk/benefit analyses.

The current study had several limitations that must be carefully considered in interpreting the study findings and/or attempting to generalize the results. The participants in this research study were recruited from a pool of individuals that participate in a virtual longitudinal study (web-based Louisiana Aging Brain Study) [19.^20^]. As such, this study was not conducted as part of routine clinical care and was not part of a geriatric assessment or fitness-to-drive assessment. It is anticipated that patients who complete the BES geriatric assessment as part of routine clinical care may be more challenged in completing the assessment because of the potential for their responses to result in the potential restriction of their driving privileges. Secondly, the current feasibility study was conducted in a participant pool that is not representative of the general population and future studies will need to be conducted in a larger and more diverse sample of drivers (sex, race, ethnicity, education, health history).

## CONCLUSIONS

The current study demonstrates the initial feasibility and validity of using a BES geriatric assessment to collect comprehensive medical and non-medical data necessary for geriatric assessments and medical fitness-to-drive evaluations in older adult drivers. This self-guided, electronic approach successfully captured data on demographics, transportation access, driving history, driving perceptions and self-restrictions, health conditions, and medication usage with minimal need for corrections to participant responses, in the current study. The BES geriatric assessment has the potential to lower the clinical burden of administering comprehensive geriatric assessments, increase patient access to fitness-to-drive evaluations, and facilitate proactive physician-patient discussions about driving safety. Future studies should validate these findings in larger, more diverse populations and explore the integration of telematics data and AI-based predictive modeling to further enhance the clinical utility of this approach.

## Supplementary Material

Table S1

Table S2

Table S3

Table S4

Table S5

The following supplementary materials are available online, Table S1: Perceptions of driving abilities, distracted driving, head/neck mobility, and reflexes, Table S2: Importance of driving to independence, QoL, and health, Table S3: Driving self-restrictions and discussing driving with physician, Table S4: Health status, medical conditions, surgery, hospitalization, and falls, Table S5: Reported prescription and over-the-counter medication use that can adversely impact driving.

## Data Availability

The authors will follow the 2003 NIH data policy for grant applications submitted prior to January 2023. Access to deidentified data outlined in this study will be available using two links: baseline BES-GA data Baseline, adjusted baseline results Adjusted Baseline. This is an original work, that the authors and participants have approved for publication. This manuscript and work outlined are not being considered for publication elsewhere and have not been published elsewhere. There are no additional permissions or reproduced copyright works related to this manuscript.
