# Supplementary material for: Initial Feasibility and Criterion Validation of an Electronic, Self-Administered, Geriatric Assessment to Facilitate Fitness-To-Drive Evaluations in Older Adults
Source: Adv Geriatr Med Res. Author manuscript; Available in PMC 2026 Jul 5. (PMC13332748; doi:10.20900/agmr20260011)
Supplement: Table S1 [file NIHMS2185830-supplement-Table_S1.pdf]

Supplementary Material

Table S1. Perceptions of driving abilities, distracted driving, head/neck mobility, and reflexes.

**Perceived driving abilities**	
Excellent	27% (13)
Very good	63% (31)
Good	10% (5)
Fair	0% (0)
Poor	0% (0)
**Easily distracted**	
Yes	10% (5)
No	90% (44)
**Use cell phone while driving**	
Yes	43% (21)
*Use phone for communication**	(18)
*Assist with navigation**	(9)
*Music/podcasts**	(4)
**Ability to move head, neck, shoulders for driving**	
Excellent	37% (18)
Very good	47% (23)
Good	10% (5)
Fair	6% (3)
Poor	0% (0)
**Reflexes as related to driving**	
Excellent	39% (19)
Very good	41% (20)
Good	18% (9)
Fair	2% (1)
Poor	0% (0)

**Note that some drivers reported more than one use of cell phone while driving.*

Table S2. Importance of driving to independence, QoL, and health.

**Importance of driving to independence**	
Essential	73% (36)
Very important	22% (11)
Important	0% (0)
Somewhat important	2% (1)
Not very important	0% (0)
**Importance of driving to QoL**	
Essential	65% (32)
Very important	27% (13)
Important	2% (1)
Somewhat important	6% (3)
Not very important	0% (0)
**Importance of driving to health**	
Essential	55% (27)
Very important	31% (15)
Important	0% (0)
Somewhat important	10% (5)
Not very important	4% (2)

Table S3. Driving self-restrictions and discussing driving with physician.

**Restricted the amount of driving they do**	
Yes	18% (9)
Has restricting amount of driving negatively impacted your life	
Yes	(2)
No	(7)
No	82% (40)
**Restricted the locations they drive**	
Yes	67% (33)
Avoid construction	6% (3)
Avoid congestion	41% (20)
Avoid rush hour	43% (21)
Avoid nighttime driving	35% (17)

Avoid inclement weather	45% (22)
No	24% (12)
**Discuss driving with physician**	
Yes	4% (2)
No	96% (47)

Table S4. Health status, medical conditions, surgery, hospitalization, and falls.

**Rating of overall health**	
Excellent	(17)
Very good	(21)
Good	(9)
Fair	(2)
Very Poor	0% (0)
**Have chronic medical condition known to impact driving**	
Yes	90% (44)
ADHD	8% (4)
Arthritis	33% (16)
Cancer	18% (9)
Chronic pain	6% (3)
Depression	24% (12)
Diabetes	12% (6)
General anxiety disorder	6% (3)
Glaucoma	6% (3)
Hearing loss	20% (10)
High blood pressure	43% (21)
Heart disease	6% (3)
High cholesterol	71% (35)
Sleep apnea	18% (9)
No	10% (5)
**Surgery**	
Yes	35% (17)
No	65% (32)
**Hospitalizations**	

Yes	4% (2)
No	96% (47)
**Fall**	
Yes	9% (4)
No	90% (44)
**Daytime Drowsiness**	
Yes	18% (9)
No	82% (41)

Table S5. Reported prescription and over-the-counter medication use that can adversely impact driving.

**Polypharmacy**	
Yes	31% (15)
No	69% (34)
**Taking prescription medications known to potentially impair driving**	
Yes	84% (41)
Allergy medications	39% (19)
Antidepressants	16% (8)
Diabetes medications	14% (7)
Muscle relaxers	4% (2)
Overactive bladder medications	7% (3)
Pain medications	16% (8)
Sleeping medications	12% (6)
**Taking over-the-counter medications known to potentially impair driving**	
Yes	20% (10)
Allergy medications	12% (6)
Sleep aids	4% (2)
No	80% (39)
